# Surgical Intervention for Penile Methamphetamine Injections

**DOI:** 10.1155/2015/467683

**Published:** 2015-09-15

**Authors:** Thomas W. Gaither, E. Charles Osterberg, Mohannad A. Awad, Benjamin N. Breyer

**Affiliations:** Department of Urology, University of California, San Francisco, 1001 Potrero Avenue, Suite 3A20, San Francisco, CA 94117, USA

## Abstract

Methamphetamine is a central nervous system stimulant and is the second most commonly used illicit drug after cannabis. Methamphetamine use for sexual pleasure is well documented. In this case report, we describe two cases presenting to our urban county hospital associated with complications related to penile injection of methamphetamine. Both patients developed penile abscesses and required urgent surgical incision and drainage. Penile abscesses represent a rare complication associated with IV drug administration into the penile corpora. Resultant penile abscesses require broad-spectrum antibiotics and surgical drainage. Further understanding of methamphetamine abuse along with the role it plays in sexual enhancement would be an invaluable addition to understanding of the rationale behind this self-administered stimulant. Drainage of penile abscesses associated with IV drug users may be hazardous to healthcare providers who are at risk from a needle stick injury.

## 1. Introduction

Methamphetamine is a central nervous system stimulant and is the second most commonly used illicit drug after cannabis [1]. The effects of methamphetamine include euphoria, arousal, increased sexual pleasure, and psychomotor agitation [2, 3]. In particular, methamphetamine use is reported to prolong sexual performance and to delay and enhance orgasm [4]. Worldwide, it is estimated that around 51 million individuals have used methamphetamine at least once in the last 12 months [1]. The usage of methamphetamine is becoming increasingly popular in urban areas among gay and bisexual men with HIV [5]. In San Francisco and Los Angeles, 11–13% of gay and bisexual men reported using methamphetamine in the past 6 months [6].

Methamphetamine use for sexual pleasure is well documented [1, 4, 7]. In addition to enhanced sexual pleasure, homosexual men have reported using methamphetamine to self-medicate the negative effects associated with HIV serostatus and may engage in hypersexual behaviors without sexual protection [8, 9]. Concomitant illicit drug use occurs frequently with methamphetamine users and is associated with high-risk sexual behaviors. As methamphetamine is commonly used with other drugs, such as cocaine and rohypnol, it has been shown to promote high-risk sexual behavior, such as sex without condoms, anonymous sex, and receptive anal sex [10, 11]. In contrast to its use for sexual enhancement, methamphetamine use has also been associated with genital self-mutilation [12].

Methamphetamine is commonly administered via the following routes: intranasal, oral ingestion, pulmonary inhalation, and IV injection [1, 3]. In this case report, we describe two cases presenting to our urban county hospital associated with complications related to penile injection of methamphetamine. Both patients developed penile abscesses and required urgent surgical incision and drainage.

## 2. Case 1

A 47-year-old man with a history of methamphetamine use, prior penile abscesses, urethral foreign body insertions, HIV, hepatitis C, and diabetes mellitus presented to the emergency department with severe penile pain, fevers, and scrotal swelling. Several days prior to admission, the patient reported injecting methamphetamine into his corpus cavernosum. He denied any fever or difficulty emptying his bladder. He reported having sex with men without prophylactic condoms. He denied other active substance abuse and/or suicidal ideation. On exam, needle fragments were identified around the abscess cavity.

**Figure 1:**
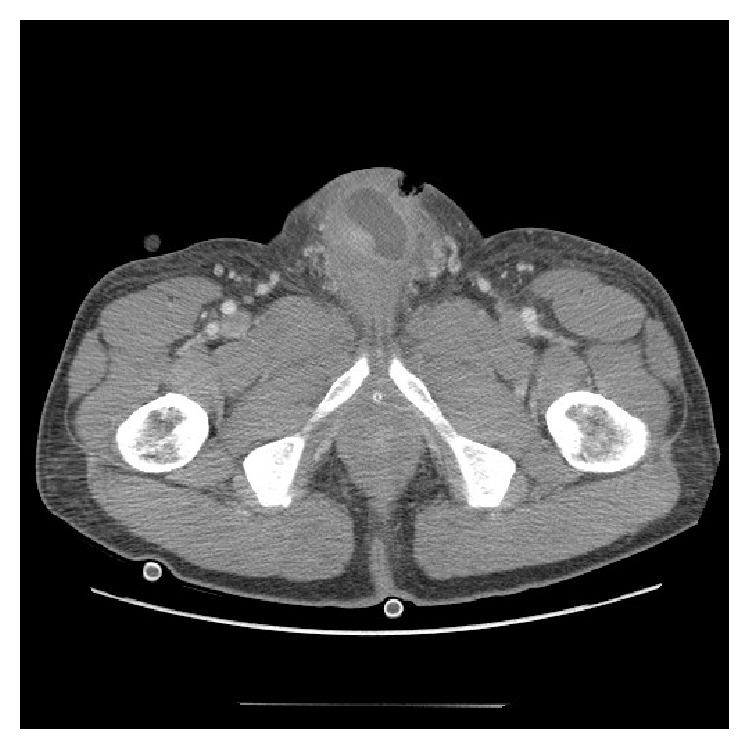
Contrast CT scan of the pelvis of Case 1. CT scan showed abscesses within or adjacent to the bilateral corpus cavernosa. The largest abscess was located in the left corpus cavernosum, measuring 4.8 × 2.8 × 4.4 cm. The additional left corpus cavernosum and right corpus cavernosum abscesses measured 2.4 cm and 1.6 cm, respectively, in longest diameter on coronal views.

Initial laboratory evaluation demonstrated a white blood cell count of 14,000/*μ*L, hematocrit of 40%, platelets of 316,000/*μ*L, creatinine of 1.01 mg/dL, and lactic acid of 0.8 mg/dL. Urinalysis was nitrate negative, positive for leukocyte esterase and had 10–20 white blood cells, 0–2 red blood cells, and squamous epithelial cells. Bedside ultrasound performed in the emergency room showed no apparent fluid collections in the penis. A contrast CT scan of the pelvis is shown in Figure 1.

The patient was taken to the operating room on the day of admission for incision and drainage of complex penile abscesses. Initial intraoperative ultrasound was performed noting a deeper fluid collection near the left proximal corpora. The abscess cavity was opened whereby 100 cc of purulent foul-smelling fluid drained spontaneously. The cavity was interrogated with a clamp and washed out with normal saline and packed wet to dry with gauze. An ultrasound of the right corporal base was performed which also demonstrated a 2 cm fluid collection, which was also drained similarly. There was no obvious involvement of the urethra.

The patient's postoperative hospital course was uneventful. He was placed on IV vancomycin (1.5 g every 8 hours) and ertapenem (1 g IV every 24 hours). The patient was discharged two weeks following initial presentation. His intraoperative wound cultures speciated with* Streptococcus viridans* spp.

## 3. Case 2

A 33-year-old male with no significant past medical history presented to the emergency department for evaluation of fevers, chills, and sharp penile pain, which began the day following a self-administered injection of methamphetamine into his penis. He denied any other significant past medical or surgical history. His family history was noncontributory. The patient endorsed a history of depression and reported having sex with only women.

**Figure 2:**
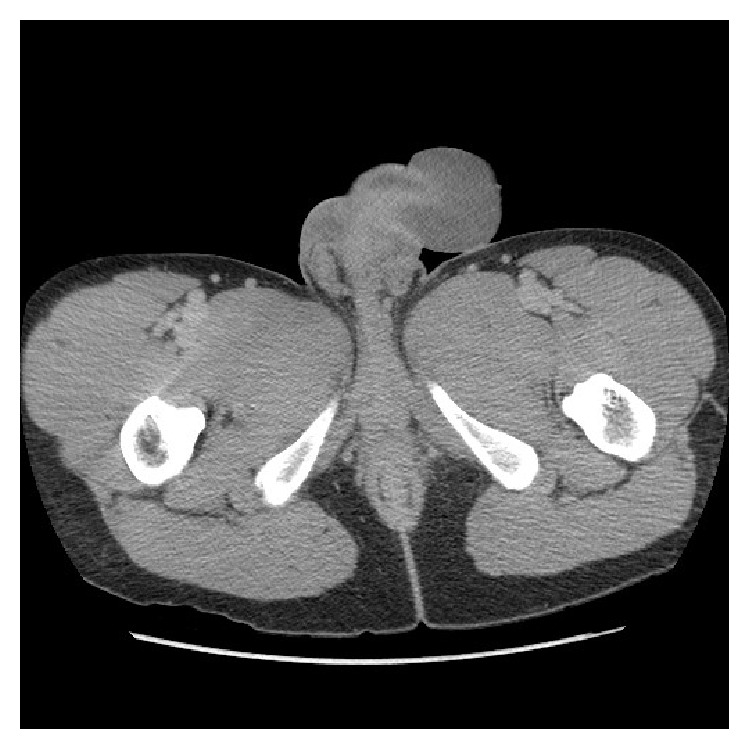
Contrast CT scan of the pelvis of Case 2. CT scan showed diffuse edematous thickening of the skin of the penis without evidence of focal fluid collection or abscess.

Laboratory studies showed a white blood cell count of 22,000/*μ*L, hematocrit of 37%, a platelet count of 374,000/*μ*L, creatinine of 1.11 mg/dL, and lactic acid of 3.9 mg/dL. CT scan of the pelvis with contrast is shown in Figure 2.

Despite negative CT imaging, the patient was immediately taken to the operating room for incision and drainage after progressively worsening in the emergency department. A 17-French flexible cystoscopy confirmed no evidence of urethral erosion, injury, or stricture. After placement of a Foley catheter, a 7 cm incision along the right lateral aspect of the penis was performed whereby pus was drained. The abscess cavity was copiously irrigated and packed wet to dry. There was no involvement of the corpora or urethra. His postoperative course was complicated by pulmonary edema managed conservatively with diuresis. Initially, he was started on vancomycin (1.5 g every 8 hours) and ertapenem (1 g IV every 24 hours) antibiotics until his intraoperative wound culture grew sensitive Group A* Streptococcus* spp. He was discharged home with amoxicillin/clavulanate 875 mg/125 mg twice daily for 14 days based off antibiotic sensitivities.

## 4. Discussion

Here we discuss two case reports of penile abscesses following intracorporal injection of methamphetamine from an urban, county hospital. Both patients underwent surgical incision and drainage in conjunction with IV antibiotics. Prior literature has reported on the penile veins, specifically the dorsal vein of the penis, being used for IV drug administration and is associated with necrotizing ulcers [13]. Prolonged IV drug users suffer from venous sclerosis and thus may resort to more dangerous sites of injection that is femoral, axillary, jugular, or penile veins. As a result from penile injections, penile gangrene has been reported [14].

In both cases, the patients had a previous history of psychiatric disorders. The psychological reasons for penile injections are unknown. The first patient identified as a man who has sex with men (MSM). The second patient identified as a man who has sex with women (MSW). The use of methamphetamine has been reported to be up to 11–13% among MSM from urban US cities [6, 15]. Although methamphetamine is often used to enhance sexual pleasure [1], no prior data suggests an association between penile injection drug use and sexual orientation. However, it has been documented that MSM who inject methamphetamine are more objectively impulsive [16].

Treatment for methamphetamine addiction is limited with no approved medication for dependence [1]. The route of drug abuse of methamphetamine does seem to impact treatment outcomes, as injectors have the poorest treatment prognosis as compared to intranasal users and smokers [2]. Although the exact psychological motivation for penile injection is unclear, more research is required, as this may become a growing trend among methamphetamine users.

The diagnosis of a penile abscess is usually clinical along with supportive imaging studies, such as ultrasound or CT scans [17]. More recent data suggest the use of magnetic resonance (MR) imaging to determine the extent of infection or inflammation, which can aid in surgical drainage of scrotal or penile pathology [18]. For diagnosing other soft tissue abscesses, ultrasound is more sensitive than CT, but CT is more specific for superficial soft tissue abscesses [19]. However, Case 2 in our report did not have radiological evidence of a penile abscess, yet pus was clearly identified in the operating room. Thus, clinicians must have a high index of suspicion for penile abscess with a patient who has a history of penile injection, along with penile pain and other signs of infection, such as fevers or a leukocytosis. MR imaging could be used if clinical symptomology is less clear [18].

Although penile abscesses are relatively uncommon, a few case reports have been reported in the literature. Common etiologies of penile abscesses include trauma, injections, iatrogenic, or idiopathic [17, 20–22]. In the cases presented, we suspect the abscesses formed as a result of direct contamination from repeated intracorporal injections. Most penile abscesses are treated with surgical incision and drainage along with antibiotic therapy [17]. Incision and drainage of abscesses, especially in patients with a history of IVDU, carry some risk of needle stick injuries to healthcare providers as a result of needle breakage [23]. Therefore, healthcare providers should avoid blunt manual dissection at the time of surgical exploration. The most common complication following penile abscesses is penile curvature from fibrosis [17, 24].

## 5. Conclusion

In summary, penile abscesses represent a rare complication associated with IV drug administration into the penile corpora. Resultant penile abscesses require broad-spectrum antibiotics and surgical drainage. Further understanding of methamphetamine abuse along with the role it plays in sexual enhancement would be an invaluable addition to understanding of the rationale behind this self-administered stimulant. Drainage of penile abscesses associated with IV drug users may be hazardous to healthcare providers who are at risk from a needle stick injury.
